# Phylogenetic Diversity in the Macromolecular Composition of Microalgae

**DOI:** 10.1371/journal.pone.0155977

**Published:** 2016-05-26

**Authors:** Zoe V. Finkel, Mick J. Follows, Justin D. Liefer, Chris M. Brown, Ina Benner, Andrew J. Irwin

**Affiliations:** 1 Environmental Science Program, Mount Allison University, Sackville, New Brunswick, Canada; 2 Department of Earth, Atmosphere and Planetary Sciences, MIT, Cambridge, MA, United States of America; 3 Department of Chemistry and Biochemistry, Mount Allison University, Sackville, New Brunswick, Canada; 4 Department of Math and Computer Science, Mount Allison University, Sackville, New Brunswick, Canada; INRA, FRANCE

## Abstract

The elemental stoichiometry of microalgae reflects their underlying macromolecular composition and influences competitive interactions among species and their role in the food web and biogeochemistry. Here we provide a new estimate of the macromolecular composition of microalgae using a hierarchical Bayesian analysis of data compiled from the literature. The median macromolecular composition of nutrient-sufficient exponentially growing microalgae is 32.2% protein, 17.3% lipid, 15.0% carbohydrate, 17.3% ash, 5.7% RNA, 1.1% chlorophyll-a and 1.0% DNA as percent dry weight. Our analysis identifies significant phylogenetic differences in macromolecular composition undetected by previous studies due to small sample sizes and the large inherent variability in macromolecular pools. The phylogenetic differences in macromolecular composition lead to variations in carbon-to-nitrogen ratios that are consistent with independent observations. These phylogenetic differences in macromolecular and elemental composition reflect adaptations in cellular architecture and biochemistry; specifically in the cell wall, the light harvesting apparatus, and storage pools.

## Introduction

Over 80 years ago Alfred Redfield discovered that plankton have an average atomic C:N:P stoichiometry of 106:16:1 [1]. The Redfield ratio is a key concept in oceanography that links nutrient availability in the ocean surface and the elemental composition of plankton to carbon storage in the ocean [2–4]. However, there is tremendous variation in C:N:P in phytoplankton. A recent global compilation found systematic geographic variability in surface ocean particulate C:N:P, with significant deviations from the Redfield ratio [5]. Laboratory studies and field analyses have identified species-level differences in C:N:P and trace element composition across phytoplankton species that reflect their evolutionary history and acclimation to environmental conditions [5–7]. It has been hypothesized that taxonomic differences in C:N:P across phytoplankton species may be responsible for geographic variation in particulate C:N:P in the sea surface [3,5]. Models show that biogeographic variation in the relative proportion of phytoplankton taxa with different average C:N:P can significantly impact the efficiency of ocean carbon storage [3]. Here, through the compilation and analysis of data from many experimental studies, we test the hypothesis that there are systematic variations in C:N in microalgae at the phylum level which are due, in part, to adaptations of cellular architecture and biochemistry that correspond to changes in macromolecular composition.

The C:N:P of microalgae reflects their macromolecular composition [8,9]. Protein is the primary functional reservoir of cellular nitrogen (N) while phospholipids and nucleic acids are the major functional reservoirs of cellular phosphorus (P). Cellular carbon (C) is largely determined by the combination of protein, lipid and carbohydrate. Macromolecular composition offers an attractive modeling framework for interpreting and predicting stoichiometry in microalgae and the biogeochemistry and biogeography of C:N:P [10] and will be useful for understanding competition and predator-prey interactions as well as developing microalgae for biotechnological applications such as the production of biofuels and nutritional supplements [9,11]. Differences in macromolecular stoichiometry and storage pools, across species and within species as a function of changes in environmental conditions, will lead to changes in the C:N:P of microalgae biomass [8]. For example, C:N in cultured microalgae often increases with nitrogen depletion as growth rate and protein content decline and carbohydrate and lipid stores increase [12]. Several different mechanisms have been proposed to link the major macromolecular pools to elemental stoichiometry and growth rate [9,10,13,14], highlighting gaps in current understanding and a need for a systematic analysis of the taxonomic and environmental variability in the macromolecular composition of microalgae.

Pioneering work by Parsons et al. [15] on 11 species of microalgae from 5 classes established that protein is 17–57% (average: 39%), carbohydrate is 4.1–37% (23%) and lipid is 2.9–18% (8.3%) of dry weight. Analogous to early work on the Redfield ratio, Parsons et al. [15] concluded that marine microalgae have similar macromolecular composition when grown under similar conditions regardless of cell size or taxonomic class. A more recent analysis of 40 species of microalgae in the late exponential phase of growth [16] and 11 species grown under semi-continuous conditions [17] also found large differences in macromolecular pools across species and no clear differences across phyla or classes. There is some evidence that Cyanobacteria may be lower in lipid as percent dry weight than other microalgae, but high levels of variability across strains has led to the hypothesis that the ability to produce large quantities of lipid may be species- or strain-specific [18].

It is difficult to reconcile invariance in macromolecular composition across phyla of microalgae with known differences in C:N:P across phyla [6,7]. Previous efforts may have failed to detect variation in macromolecular composition due to insufficient data, the amount of variation within species swamping the variation among phyla, and the challenge of analyzing variation with unbalanced sampling across taxonomic categories. Since Parsons et al. [15] more than 100 studies have quantified the macromolecular composition in many dozens of species of microalgae. Here we compile macromolecular composition data from 130 studies and use a hierarchical Bayesian analysis to determine the median macromolecular composition of microalgae and test if there are phylogenetic differences across phyla. The hierarchical Bayesian model has significant advantages compared to other statistical methods. The hierarchical structure of the model accommodates widely varying numbers of observations per species, allowing each observation to contribute to the grand and phylum means or medians without the uneven sampling distorting the means or medians or size of the credible intervals.

## Materials and Methods

### The Microalgae Macromolecular Database

Macromolecular data for microalgae, predominantly marine phytoplankton, was collected from 130 publications from tables, text and figures. The microalgae macromolecular database and list of data sources is available in S1 Table and S1 File. No exclusion terms were used when searching for publications. Many of the papers were found by searching Google Scholar from 2013–2015 using the terms phytoplankton, algae, or microalgae and protein, lipid, carbohydrate, ash, RNA and DNA. Data from figures was captured using ImageJ software. Macromolecular composition (protein, carbohydrate, lipid, chlorophyll a, RNA and DNA) as mass per cell and as percent dry weight was recorded along with the taxonomic information (phylum, genus, species and strain information), culture conditions (semi-continuous culture, turbidostat, chemostat, batch culture), and growth phase (lag, exponential or stationary phase of the batch culture). For this analysis we focused on 222 marine and freshwater microalgae species with 971 observations under exponentially growing nutrient-sufficient conditions in batch, turbidostat and semi-continuous cultures. For comparison we computed the macromolecular composition of 117 species of microalgae with 591 observations in the stationary phase of growth. In total this includes 751 estimates of cellular protein and 461 estimates of protein as percent dry weight, 575 and 436 estimates of cellular carbohydrate and percent carbohydrate, respectively and 502 and 699 estimates of cellular and percent lipid, respectively. There are many fewer studies and observations of nucleic acid content (RNA and DNA). To take advantage of as much data as possible the ratio of protein to carbohydrate, protein to lipid and carbohydrate to lipid was calculated for any species within a study under the specified experimental conditions (e.g., under nutrient-sufficient exponential growth in batch, turbidostat, and semi-continuous culture) whether it was expressed on a mass per cell or percent dry weight basis. Some species, especially those used in aquaculture and those that are considered candidates for the biofuel industry are over-represented in the database. In particular, *Isochrysis* (n = 194, where n refers to the total number of observations), several species of *Chaetoceros* (n = 129), *Thalassiosira* (n = 113), *Tetraselmis* (n = 71), *Chlorella* (n = 51), *Dunaliella* (n = 62) and *Nannochloropsis* (n = 57) have been studied by numerous groups under a range of culture conditions.

AlgaeBase an online database of terrestrial, marine and freshwater algae (http://www.algaebase.org/) was used (2014–2016) to identify synonyms, phyla and if species were freshwater, terrestrial or marine. Species listed as coastal, estuarine or brackish were considered marine. Species from 9 phyla, including the Cyanobacteria, Chlorophyta, Rhodophyta, Bacillariophyta, Cryptophyta, Dinophyta, Euglenozoa, Haptophyta, and Ochrophyta, were collected but the majority of observations are from species within the Bacillariophyta followed by the Haptophyta and Chlorophyta. Very few observations were obtained for the Euglenozoa and Rhodophyta and therefore these data are not used in analyses that compare phyla but are included in the pan-microalgae estimates of macromolecular pools and ratios. Species identified to the genus but not species level were assumed to be different species unless identified as the same strain within or across studies. The majority of observations in the database are from marine species. Under active growth conditions, for protein as percent dry weight, 79% of the observations are marine, for carbohydrate as percent dry weight 80% of the observations are marine, and for lipid as percent dry weight 75% of the observations are marine. All observations for the Cryptophyta and Dinophyta are marine, most of the macromolecular observations for Haptophyta and Ochrophyta are marine, >60% of the macromolecular observations for Chlorophyta are marine, but for the Cyanophyta most of the observations (>60%) are from freshwater species.

### Protein data

Several methods are used to determine protein content in microalgae. Total nitrogen content (N content) can be measured, often using the Kjeldahl method, and then converted to protein using a conversion factor, or protein can be estimated from peptide residues (Lowry, Bicinchonicic acid, or Bradford assays), or amino acids can be measured and summed. Most of the protein estimates in the database used a Lowry-type assay or N content. Traditionally N content is converted to protein assuming protein is 16% N by mass and all measured N is in protein. The largest non-protein nitrogen pool in microalgae is associated with inorganic nitrogen pools in eukaryotic species [19]. Cyanobacteria tend to store nitrogen as protein and peptides. We therefore corrected protein values derived from total nitrogen content for non-protein nitrogen in the eukaryotic species following Lourenço *et al*. (2004). We then used all estimates of protein to estimate phylogenetic differences in macromolecular composition but excluded protein data estimated from N content for all comparisons to C:N data. A comparison of protein as a percentage of dry weight for exponentially growing microalgae for the different methods is provided in Table 1.

**Table 1:** Protein content estimates (% dry weight) by phylum and grand mean over all groups. Protein observations are grouped by method: assays that measure amino acids and peptide residues (Amino acid and peptide residues) and measurements of nitrogen that are converted to protein (N content) using a conversion factor (6.25) based on the assumption that protein is 16% nitrogen by mass. We apply the correction factor of 4.78 g protein / g N instead of the standard conversion of 6.25 g protein / g N as recommended by Lourenço et al (2004) for all the eukaryotic phyla to account for non-protein nitrogen (Corrected N content) and then pool the corrected N content derived protein observations with amino acid and peptide based estimates of protein into a pooled protein estimate. The pooled estimate can be larger or smaller than all of the first three columns because of the hierarchical pooling of data (see Methods). The top value is the median percent dry weight, the middle values in brackets denote the 95% credible interval on the median, and the bottom value is the number of observations.

Phylum	Amino acids and peptide residues	N content	Corrected N content	Pooled protein estimate
Cyanobacteria	42.2	41.3		43.2
	(32.9, 50.4)	(33.9, 49.4)		(37.1, 49.3)
	19	6		25
Chlorophyta	32.8	42.6	33.0	32.7
	(28.5, 37.2)	(38.1, 47.5)	(29.1, 37.3)	(29.3, 36.1)
	51	24	24	75
Cryptophyta	37.7	45.0	35.3	38.4
	(28.2, 50.5)	(38.0, 53.5)	(28.8, 42.6)	(31.1, 46.2)
	5	11	11	16
Bacillariophyta	29.2	35.2	26.5	27.4
	(24.2, 33.8)	(30.4, 40.1)	(22.6, 30.8)	(24.0, 31.0)
	46	36	36	82
Haptophyta	32.5	41.0	31.8	32.0
	(26.1, 38.7)	(36.3, 46.2)	(27.4, 36.2)	(27.4, 36.4)
	40	36	36	76
Ochrophyta	32.8	40.5	31.6	32.5
	(24.5, 41.2)	(31.0, 51.3)	(21.3, 40.5)	(25.4, 39.9)
	18	1	1	19
Dinophyta	30.4	37.5	28.2	27.4
	(19.1, 39.0)	(26.8, 45.7)	(18.4, 35.7)	(19.2, 34.9)
	20	2	2	22
Grand mean	32.7	40.2	31.2	32.2
	(30.2, 35.0)	(38.0, 42.6)	(29.3, 33.3)	(30.4, 34.0)
	199	118	118	317

### Bayesian analyses of the average macromolecular composition of microalgae

We computed the median macromolecular composition as percent dry weight and the mass ratios of macromolecular pools for nutrient-sufficient exponentially growing cultures using a hierarchical Bayesian model [20]. For comparison we computed the median macromolecular composition normalized to dry weight from the stationary phase of batch culture. The literature survey resulted in a very unbalanced design, with some species and phyla having many observations and others very few. Our approach was designed to incorporate all the available data without allowing unbalanced sampling (for example different numbers of observations within phyla or disproportional sampling of certain species) to distort the estimates. We expressed each quantity as a sum of random variables for each species, each phylum, and an overall mean,

$$y_{i}=\mu +P_{p\left[i\right]}+S_{s\left[i\right]}+e_{i}$$ (1)

where *y*_i_ are the observations, *μ* is the overall mean, *P*_j_ and *S*_j_ are the estimated means for each phylum and species, respectively, *p*[*i*] and *s*[*i*] are the phylum and species, respectively, of observation *i*, and *ε*_i_ is the residual error for each observation. Each of the estimates (*μ*, *P*_j_, *S*_j_) was described by a normal distribution. Three distinct uninformative hierarchical priors were used for the variances of species and phylum means and the error term [21, Section 5.2]. The hierarchical model has the effect of partially pooling the data across taxonomic levels, sharing the sampling strength across taxa and leading to smaller variances than would be obtained in a classical regression. This pooling can lead to apparent discrepancies, for example in the protein method analysis (Table 1) the protein estimate for some phyla from the full dataset can be either larger or smaller than both the N content and protein content method estimates. These results represent our best estimates and the apparent inconsistency is a result of pooling combined with small sample sizes, the distribution of observations among species, and relatively large uncertainties. This model does not identify the overall mean so we computed the overall mean from phylum means weighted by their inverse variances. We report the posterior median of each variable, but the posterior uncertainties of many of the species medians were very large so species level results are not reported. We choose to report the median because it provides a measure of central tendency that is less sensitive to a skewed distribution than the mean. The ratios of macromolecular pools are modeled in the same way except we modeled the log of the ratios and report the inverse-log transformed results. C:N were computed from the percent contribution of six macromolecular pools (protein, lipid, carbohydrate, RNA, DNA, and chlorophyll a) and the chemical data in Geider and LaRoche [8]. The lipid pool was divided into two subpools: a phosphorus-free pool (2/3 of total lipid) and a phospholipid pool (1/3 of total lipid). Since most species lacked one or more of the major macromolecular pools, C:N was computed using the estimated phylum-level median macromolecular percent content (the *P*_p[i]_ for each macromolecular pool). We computed 50,000 iterations on 4 chains using Rstan and sampled the distribution from the second half of the iterations thinning the samples to every fifth observation [21]. We used the estimated standard deviations for each variable to partition the total variance into within species, within phyla and among species, and among phyla variance. To test for significant differences in both macromolecular composition (percent dry weight) and ratios, we used the empirical posterior distribution of the differences between all phyla-level medians to construct 95% credible intervals on the differences. We defined the credible intervals using the highest density interval [22]. Consistent with this analysis we did not perform classical null-hypothesis significance testing, but instead interpreted the phyla level means as different if the 95% credible interval of their difference does not overlap zero. We calculated a mean and 95% confidence interval for C:N for Cyanobacteria, Chlorophyta, Bacillariophyta and Dinophyta from published culture experiments [6,7], independent from the macromolecular database. We compared phylum level C:N estimated from the major macromolecular pools (median and 95% credible interval) with this independent phylum level estimate of C:N.

## Results

### Median macromolecular composition of microalgae

Under nutrient-sufficient growth conditions the median macromolecular composition of microalgae is 32.2% protein, 17.3% lipid, 15.0% carbohydrate, 17.3% ash, 5.6% RNA, 1.1% chlorophyll-a and 0.98% DNA as percent dry weight (Tables 2 and 3). On average these 7 components account for 90% of the dry mass of a cell. The protein to carbohydrate ratio is 2.4, the protein to lipid ratio is 2.2, and the carbohydrate to lipid ratio is 0.90 (Table 3). The ash fraction (the inorganic residue that remains after the sample is combusted) is predominately P, S, Na, Cl, K, Ca, Mg. In the Bacillariophyta and calcified microalgae, Si and Ca, respectively, are significant components of the ash. Under the stationary phase of growth in batch culture the average protein as percent dry weight declines to 27.0%, carbohydrate increases to 21.8% and lipid to 22.5%. There no significant difference in ash, chlorophyll-a, or nucleic acid content as percent dry weight between active growth conditions and stationary phase (Table 2), although there is insufficient RNA and DNA data from the stationary phase of growth to draw conclusions. The median protein, carbohydrate, ash, chlorophyll-a and nucleic acids as percent dry weight in this study is consistent with earlier work, but lipid as percent dry weight is on the higher end of observations reported in previous compilations [15,16]. The higher median lipid content in our study could be due to a shift in methods and an increased focus on oleaginous species over time [18]. The pinacyanol method used in Parsons et al. (1961) can provide extremely low lipid estimates relative to gravimetry and more recent colorimetric methods.

**Table 2:** Median macromolecular composition as percent dry weight of microalgae under nutrient-sufficient exponential growth and under the stationary phase of growth (this study) compared to the median macromolecular composition of marine bacteria and marine yeast [23], various herbaceous plants and leaves (raw spinach, green leaf lettuce, fresh spearmint, coriander, fresh basil, fresh rosemary, raw broccoli leaves, wild rhubarb leaves, winged bean leaves, raw pumpkin leaves, chrysanthemum leaves) [24], and raw chicken egg [24].

	Microalgae (active growth)	Microalgae (stationary phase)	Bacteria (n = 7)	Yeast (n = 8)	Herbs and leaves (n = 11)	Raw egg
Protein	32.2	27.0	48	32.0	27.3	52.7
	(30.4, 34)	(24.2, 29.8)				
	317	144				
Lipid	17.3	22.5	5.4	4.6	5.1	39.9
	(16.2, 18.2)	(20.7, 24.4)				
	375	324				
CHO	15.0	21.8	5.6	24.0	56.3	3.0
	(13.7, 16.5)	(18.5, 24.8)				
	308	128				
Ash	17.3	18.6	29	8.6		
	(15.4, 18.7)	(14.9, 21.7)				
	185	60				
RNA	5.65	9.9*	4.4	4.8		
	(4.64, 6.96)					
	24					
Chl-a	1.13	0.87				
	(0.9, 1.3)	(0.56, 1.19)				
	104	40				
DNA	0.98	0.6*	0.33	0.1		
	(0.25, 2.0)					
	20					
SUM	89.6	101.3	92.7	74.0	88.7	95.6

*There are only two observations of RNA and DNA in stationary phase in the database. The top value in the first 2 columns is the median percent dry weight, the middle values in brackets denote the 95% credible interval on the median, and the bottom value is the number of observations.

**Table 3:** Taxonomic differences in median macromolecular composition as percent dry weight under nutrient-sufficient exponential growth conditions. The top value is the median percent dry weight, the middle values in brackets denote the 95% credible interval on the median, and the bottom value is the number of observations.

	Protein	Lipid	CHO	Ash	RNA	Chl a	DNA
Cyanobacteria	43.1	11.7	21.8	8.12	8.7	1.06	0.82
	(36.8, 49.3)	(8.23, 16.2)	(16.7, 26.2)	(4.88, 10.9)	(7.28, 10.1)	(0.54, 1.4)	(0.63, 4.7)
	25	33	22	13	16	3	16
Chlorophyta	32.7	16.3	14.4	12.1	5.11	1.15	0.81
	(29.4, 36.1)	(14.5, 18.2)	(11.5, 16.8)	(6.45, 14.2)	(2.79, 8.22)	(0.90, 1.5)	(0.40, 6.2)
	75	98	71	33	3	26	3
Cryptophyta	38.5	16.1	12.5	16.1		1.18	
	(30.8, 45.8)	(11.2, 20.3)	(7.03, 17.8)	(10.2, 19.5)		(0.85, 1.6)	
	16	18	16	13		8	
Bacillariophyta	27.4	18.8	12.2	27.5		1.12	
	(23.9, 30.8)	(16.9, 20.8)	(9.57, 15)	(24.1, 29.3)		(0.84, 1.4)	
	82	92	82	63		23	
Haptophyta	32.1	18.6	16.9	13.7	4.78	1.16	1.01
	(27.5, 36.8)	(15.7, 21.3)	(11.4, 20.2)	(11.3, 17.5)	(0, 10.7)	(0.89, 1.5)	(0.19, 1.6)
	76	81	73	51	1	34	1
Ochrophyta	32.6	21.3	14.4	19.6	0.55	1.1	
	(25.2, 39.5)	(17.1, 26.1)	(8.62, 20)	(10.7, 22.8)	(0, 3.86)	(0.70, 1.5)	
	19	26	19	8	4	10	
Dinophyta	27.4	15.8	23	11.6			
	(19.4, 35)	(12.4, 20.2)	(15.6, 29.8)	(4.16, 19.9)			
	22	25	23	2			
Grand Mean	32.2	17.3	15	17.3	5.65	1.13	0.98
	(30.4, 34)	(16.2, 18.2)	(13.7, 16.5)	(15.4, 18.7)	(4.64, 6.96)	(0.9, 1.3)	(0.25, 2.0)
	317	375	308	185	24	104	20

### Taxonomic variability in the macromolecular composition of microalgae

**Fig 1:**
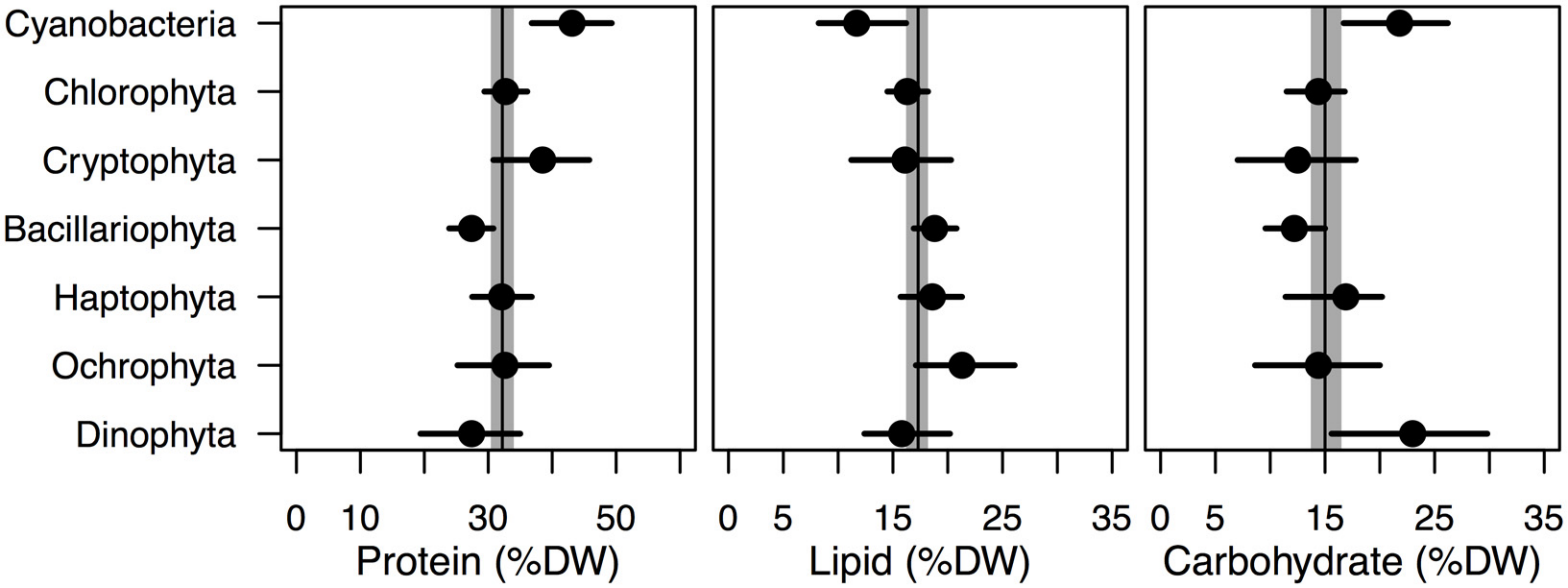
Median (filled circle) and 95% credible intervals (line) for protein, lipid and carbohydrate as percent dry weight for different phyla of microalgae under nutrient-sufficient exponential growth. The median protein, lipid and carbohydrate as percent dry weight and associated 95% credible interval across all phyla are represented by the vertical black line and grey region, respectively.

**Fig 2:**
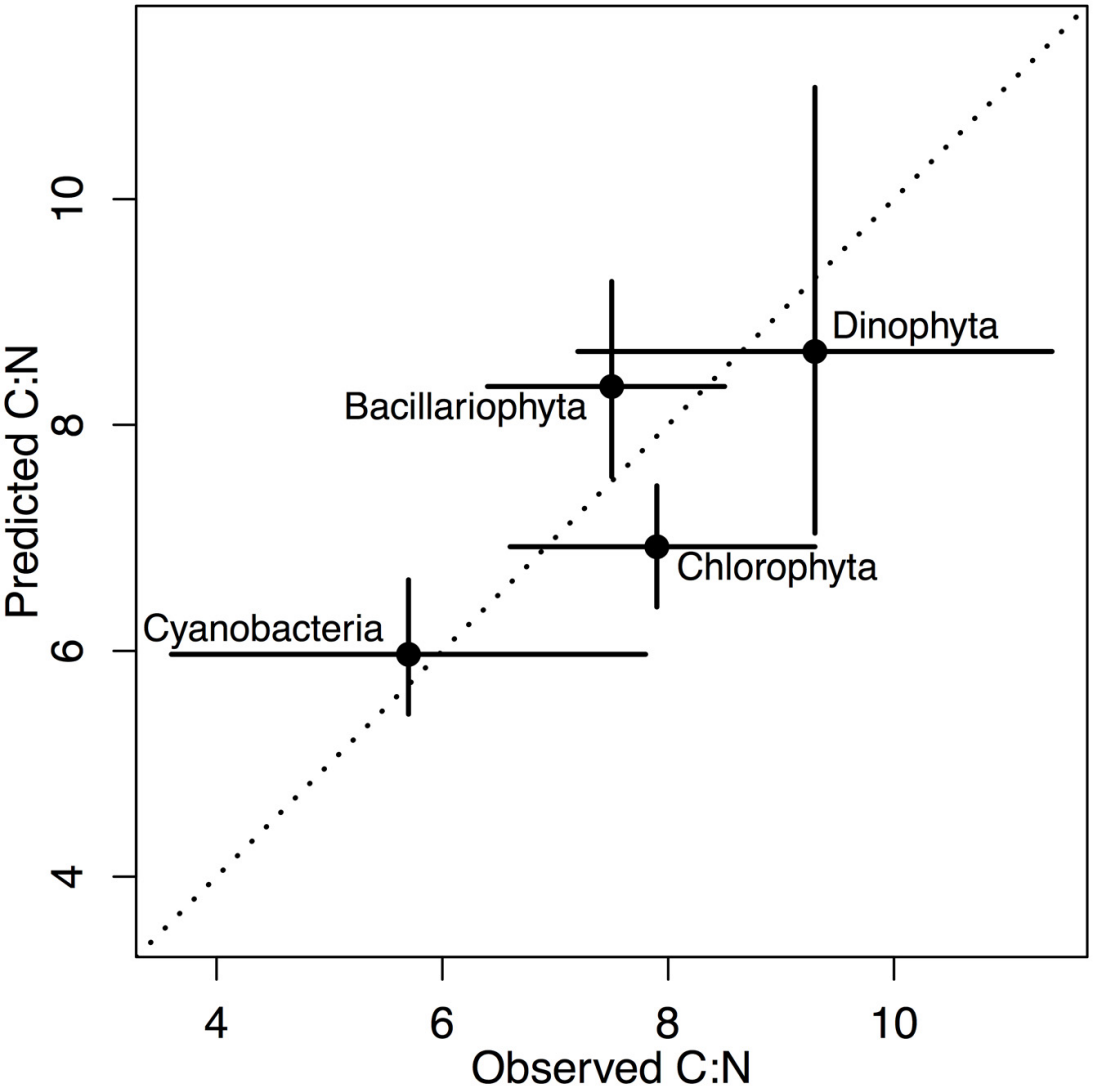
The carbon-to-nitrogen ratio in microalgae calculated from the median macromolecular composition (Predicted C:N) compared to measurements made on laboratory cultures, not associated with the macromolecular database (Observed C:N). 1:1 line (dotted).

There are significant differences in the major macromolecular pools, protein, lipid and carbohydrate, across the different phyla of microalgae (Fig 1, Tables 4 and 5). There is not enough data available in our database for a robust taxonomic comparison of nucleic acid or chlorophyll-a content. Much of the variability in the major macromolecular pools under nutrient-sufficient exponential growth conditions is found within species across the different studies, ranging from 41–43% across protein, lipid and carbohydrate as percent dry weight (residual error, Table 5). An additional 31–32% of the variability in the major macromolecular pools is found across the species within phyla (% among species, within phyla) and the remainder of the variability, 23–25%, is found across the phyla (% among phyla). Much of the variability across methods and analysts will be placed within species since many species appear in multiple studies. There is phylum-level variation in protein:lipid and carbohydrate:lipid but protein:carbohydrate does not vary significantly across phyla (Table 4). Phylogenetic differences in macromolecular stoichiometry predict phylum level differences in C:N that are consistent with experimental observations of C:N. The Cyanobacteria have the lowest and the Dinophyta the highest C:N (Fig 2, Table 4).

**Table 4:** Protein:carbohydrate (Protein:CHO), protein:lipid (Protein:Lipid), and carbohydrate:lipid (CHO:Lipid) and predicted C:N (mol:mol) based on macromolecular composition for different phyla of microalgae under nutrient-sufficient exponential growth. The top value is the median macromolecular ratio, the middle values in brackets denote the 95% credible interval on the ratio, and the bottom value is the number of observations. The last column is an independent estimate of mean molar C:N measured in exponentially growing laboratory cultures [6,7], the middle values in brackets denote the 95% confidence interval, and the bottom value is the number of experimental observations.

	Protein: CHO	Protein: Lipid	CHO: Lipid	C:N (predicted)	C:N(observed)
Cyanobacteria	2.4	4.2	2.3	6.0	5.7
	(1.8, 3.1)	(3.0, 5.8)	(1.5, 3.5)	(5.4, 6.6)	(3.6, 7.8)
	23	24	22		9
Chlorophyta	2.4	2.2	0.9	6.8	7.9
	(2.0, 3.0)	(1.8, 2.7)	(0.7, 1.2)	(6.2, 7.4)	(6.6, 9.3)
	84	81	80		15
Cryptophyta	2.8	2.7	0.6	7.0	
	(2.1, 5.0)	(1.7, 4.3)	(0.4, 1.1)	(5.9, 8.2)	
	23	18	18		
Bacillariophyta	2.4	1.7	0.6	8.3	7.5
	(2.0, 2.9)	(1.2, 2.0)	(0.5, 0.7)	(7.6, 9.3)	(6.4, 8.5)
	166	132	132		12
Haptophyta	2.3	1.8	0.8	7.4	
	(1.8, 2.9)	(1.4, 2.3)	(0.6, 1.1)	(6.5, 8.5)	
	119	113	111		
Ochrophyta	2.4	1.3	0.5	8.3	
	(1.8, 3.5)	(0.9, 1.9)	(0.3, 0.8)	(7.0, 9.8)	
	40	40	40		
Dinophyta	2.2	2.3	1.6	8.6	9.3
	(1.6, 2.8)	(1.7, 3.1)	(1.0, 2.4)	(7.0, 11.0)	(7.2, 11.4)
	45	36	37		9
Grand Mean	2.4	2.2	0.9	7.0	
	(2.1, 2.7)	(2.0, 2.5)	(0.8, 1.0)	(6.7, 7.3)	
	502	446	442		

**Table 5:** Variance decomposition for median protein, lipid and carbohydrate as percent dry weight for nutrient-sufficient exponentially growing cultures into % within species (residual error), % among species within phyla and % among phyla.

	% within species (residual error)	% among species, within phyla	% among phyla
**Protein**	42	31	25
**Lipid**	43	32	23
**CHO**	41	32	25

Compared to the eukaryotic microalgal phyla the Cyanobacteria have high protein (43.1%) and carbohydrate (21.8%) and low lipid (11.7%) and ash (8.1%) as percent dry weight (Fig 1) and the highest protein:lipid and carbohydrate:lipid observed (Table 4). The Cyanobacteria are significantly higher in percent protein than the Chlorophyta, Bacillariophyta, Haptophyta, Ochrophyta and Dinophyta, and significantly higher in percent carbohydrate than the Chlorophyta, Cryptophyta and Bacillariophyta, and significantly lower in percent lipid than the Chlorophyta, Bacillariophyta, Haptophyta and Ochrophyta. The Cryptophyta are unique among the eukaryotic phyla having both high percent protein (38.5%) similar to the Cyanobacteria, but unlike the Cyanobacteria they are lower in carbohydrate (12.5%) as percent dry weight and have the highest protein:carbohydrate of all the groups examined. The Cryptophyta have significantly higher protein as percent dry weight than the Bacillariophyta and significantly lower carbohydrate as a percent dry weight than the Dinophyta.

The Bacillariophyta, Ochrophyta, and Haptophyta (all members of the Stramenopiles) are generally higher in lipid (18.6–21.3%) than many of the other phyla examined. The Ochrophyta, dominated in this study by the class Eustigmatophyceae, are particularly high in lipid (21.3%) and the Bacillariophyta are particularly low in carbohydrate (12.2%) as percent dry weight (Fig 1). The Bacillariophyta have higher ash content as percent dry weight (27.5%) than all the other phyla examined. As a result, on a percent ash-free dry weight basis the macromolecular content for the Bacillariophyta will increase relative to the other groups (Table 3).

The Dinophyta differ from the other eukaryotes in being low in protein and high in carbohydrate as percent dry weight (Fig 1, Table 3). Carbohydrate as percent dry weight in the Dinophyta is significantly higher than in the Chlorophyta, Cryptophyta and Bacillariophyta. As a result the Dinophyta protein:carbohydrate is the lowest (2.2) and carbohydrate:lipid (1.6) is among the highest of all the eukaryotic microalgae groups examined (Table 4).

There is no significant difference in the overall protein, carbohydrate or lipid as percent dry weight across the marine and freshwater species. At the phylum level, the only significant difference between freshwater (19.7%, CI: (16.1, 23.5), n = 27) and marine species (14.6%, CI: (11.0,16.7), n = 45) is in lipid as a percent of dry weight in the Chlorophyta. This is likely due to a few marine species with relatively low lipid and a few freshwater species with relatively high lipid as percent dry weight within the database as opposed to a systematic difference in lipid content between marine and freshwater species. *Botryococcus braunii*, a freshwater species primarily studied for biofuel applications, has the highest lipid as percent dry weight, 43%, while *Tetraselmis suecica* and *Dunaliella tertiolecta*, marine species, have several observations across several studies with lipid as percent dry weight ≤10%.

## Discussion

### The average macromolecular stoichiometry of microalgae

The macromolecular composition of actively growing nutrient-sufficient microalgae is distinct from other major domains of life (Table 2). The microalgae are most similar in protein and carbohydrate content to yeast and bacteria, other single-celled organisms that lack differentiated structures, than the protein- and lipid-rich animals or carbohydrate-rich plants. Even excluding woody tissues, plant vegetative tissue is more carbohydrate-rich than microalgae. The eukaryotic microalgae differ from yeast and bacteria in having higher lipid content (Table 2). Cyanobacteria are intermediate in lipid content, lower than the eukaryotic microalgae and higher than many bacteria [23]. Lipid is a space-efficient carbon and photosynthetic energy store [25] that decreases cellular density and increases buoyancy [26], so elevated lipid content may be advantageous for a planktonic lifestyle, particularly in larger eukaryotic species and species with mineralized cell walls. In the microalgae, nutrient starvation stimulates a decrease in protein and increase in lipid and carbohydrate as percent dry weight (Table 2), indicating a higher protein demand for exponentially growing cells and an accumulation of carbohydrate and lipid stores under nutrient starvation [27].

### Phylogenetic differences in the macromolecular composition of microalgae

There are clear differences in macromolecular composition and stoichiometry across phyla of microalgae (Fig 1, Table 3). These evolutionary differences in macromolecular composition may be the basis for the biogeography of different phytoplankton types and biogeochemical patterns in particulate C:N:P across environments. In contrast to previous studies we are able to detect phylogenetic differences in macromolecular composition despite large inherent variability in macromolecular pools at the species level due to a combination of a larger data set and a hierarchical Bayesian analysis. Fundamental differences in cellular architecture that define the phyla appear to dictate differences in macromolecular and elemental composition.

### Protein

Phylogenetic differences in protein content reflect differences in cell wall composition, the light harvesting apparatus, and storage reserve strategies. For example the phyla with the highest protein as percent dry weight, the Cyanobacteria and Cryptophyta use protein as an integral part of their cell wall. The Cyanobacteria have a peptidoglycan layer of sugars and small peptide chains of amino acids in their cell walls and the Cryptophyta have an outer proteinaceous pellicle [28]. In addition the Cyanobacteria (an exception being the genus *Prochlorococcus* [29]) and Cryptophyta use nitrogen-rich phycobilisomes or phycobiliproteins as part of their light harvesting apparatus. It is estimated that phycobiliprotein-containing Cyanobacteria contain 6 to 16 kg protein per mol chromophore, in contrast, eukaryotic algae without phycobiliproteins typically contain 2 to 6 kg protein per mol chromophore [30]. Cyanobacteria are also known to store nitrogen as cyanophycin (L-aspartic acid) granules and can use their phycobilisomes as a nitrogen source for growth [31]. Species with high growth rates may have higher protein levels than slower growing species. The low levels of protein in the Dinophyta are consistent with their relatively low growth rates compared to other microalgae phyla [32]. Although the often fast-growing Bacillariophyta also have low protein as percent dry weight this is due to the weight of their siliceous frustule, they are relatively high in protein (and lipid) on an ash-free dry weight basis.

### Carbohydrate

Taxonomic differences in carbohydrate content are consistent with known differences in cell wall composition. The cell wall of Cyanobacteria is rich in carbohydrate: the peptidoglycan layer is tightly bound to polysaccharides and many species have an outer membrane of lipopolysaccharides and some protein, and polysaccharide-rich sheath layers that surround the outer membrane [33]. The high carbohydrate content of the Dinophyta is likely due to their characteristic microfibrillar plates ((C_6_H_10_O_5_)_n_) that can develop below the outermost plasma membrane [34]. The Chlorophyta are intermediate in carbohydrate and protein as percent dry weight, perhaps due to the large range of cell wall types within the phylum. Many of the Chlorophyta examined have polysaccharide-rich cell walls, that include cellulose, and similar to plant walls can contain significant amounts of hydroxyproline-rich glycoprotein [35]. Some species within the Chlorophyta are naked, including *Ostreococcus*, *Micromonas*, and some *Dunaliella*, while others such as *Tetraselmis* have relatively thick cell walls composed of organic scales that coalesce forming a solid cell covering [36,37]. The cell wall can account for a significant amount of cellular mass and impact C:N:P of algal biomass; for example it has been estimated that the cell wall of some *Chlorella* species may contribute up to 22% of the dry weight of the cell [38] and wall-less mutants of *Chlamydomonas* can have C:P 14-times lower than the comparative wild-type with a wall [39]. The Bacillariophyta and Cryptophyta have the lowest carbohydrate content as percent dry weight because the cell wall of the Bacillariophyta is constructed of hydrated Si with only small amounts of tightly bound carbohydrate and protein [40] and the Cryptophyta wall is proteinaceous [28]. It has been hypothesized the siliceous frustule may have a lower cost of synthesis than non-silicified walls under some circumstances [41].

### Lipid

Phylogenetic differences in lipid as percent dry weight appear to be due to differences in cell wall composition and investment in storage lipid. The Cyanobacteria have lower lipid content (11.7% of dry weight) than the eukaryotic microalgae. Most bacteria accumulate glycogen or poly3-hydroxybutrate (PHB) or other polyhydroxyalkanoates (PHAs) as energy stores [42,43]. Some bacteria, including cyanobacteria can produce small droplets (30 to 300 nm) of neutral lipids at the cell or thylakoid membrane [44]. In contrast, many eukaryotes form lipid bodies of triacylglycerides (TAGs) at the endoplasmic reticulum that can range in size from 0.1 to 50 μm in diameter [43]. The phyla with the highest lipid as percent dry weight (18.6–21.3%), in particular the Bacillariophyta, Haptophyta and the Ochrophyta, are known to produce significant lipid stores, especially under nutrient-starvation [18]. These results are consistent with the hypothesis that larger microalgae can accumulate larger lipid stores that provide a growth advantage under variable resource supply [45].

### The other macromolecular pools

The Bacillariophyta, due to their siliceous frustules, have the highest and the Cyanobacteria have the lowest ash content as percent dry weight. There is currently not enough information available to determine if there are phylum level differences in RNA and DNA content as a percentage of dry weight (Table 3). Previous compilations of DNA content, from a wide range of eukaryotic taxa, indicate that DNA content is a linear function of cell size [46]. There is much less quantitative data on RNA content, although there is evidence that cellular content increases with growth rate [9,47]. Quantitative estimates of RNA content can be difficult to obtain due in part to RNA’s susceptibility to degradation and some of the older methods (orcinol) can overestimate RNA content due to interference by other sugars [47]. Our compilation did not focus on pigment data and we do not have enough data to do a taxonomic comparison. The average estimate of chlorophyll-*a* content, 1.1% dry weight, is consistent with previous work, but both the ratio of chlorophyll-*a* to other pigments and cellular content will vary with irradiance and can reach much higher values under low irradiance [8].

### Sources of variability and potential biases

The compilation of macromolecular data from the literature and hierarchical Bayesian analysis allows us to use data generated from many labs, increasing sample size and taxonomic breadth, to discover phylum-level differences in macromolecular composition despite immense species-specific variability. Along with the advantages of compiling data from the literature are the disadvantages associated with experimental variability. Consistent extraction efficiency across the macromolecular pools, across species, and across labs cannot be assured. Several different methods are used to extract macromolecular pools and there is no way to quantify differences in extraction efficiency across studies in the literature. Methods and standards used to quantify the macromolecular pools also differ across studies. For example there are five common methods used to estimate cellular protein: total particulate nitrogen can be converted to protein using a conversion factor, protein can be determined from peptide residues using the Lowry, Bicinchonicic acid, or Bradford assays, and amino acids can be measured and summed. Each method has its own biases. The Coomassie brilliant blue dye used in the Bradford assay binds disproportionately to arginine and to a lesser degree for a number of other amino acids, and the Lowry method is disproportionally sensitive to tryptophan and tyrosine [48]. The total particulate nitrogen methods often use a conversion factor that assumes all particulate nitrogen is protein. We found that N content based estimates of protein were systematically higher (7.5%) than peptide based estimates of protein across the microalgae (Table 1), primarily due to inorganic nitrogen stores within the eukaryotic phyla [19]. Based on these findings we corrected the eukaryotic N content-based estimates of protein for non-protein nitrogen following Lourenço *et al*. (2004). Most studies in the database used the phenol sulfuric acid (PSA) method for the determination of carbohydrate, although increased sensitivity can be achieved with the less commonly employed 2,4,6-tripyridyl-s-triazine (TPTZ) and 3-methyl-2-benzo thiazoline hydrazine hydrochloride (MBTH) assays [49,50]. Carbohydrate estimated using the anthrone reaction is often low because the method does not detect hexosamines or mannitol and does not quantitatively detect pentoses and hexuronides [51]. Most microalgal studies extract lipid following a modified version of the Bligh and Dyer protocol [52] although the Folch method [53] often results in a better extraction, especially from lipid-rich samples [54]. Macromolecular pool estimates are also influenced by the standard used: glucose is the most commonly used carbohydrate standard, bovine serum albumin (BSA) and bovine gamma globulin (BGG) are the most common protein standards, and a large range of lipid standards have been employed, including oil mixtures as well as single fatty acids. There can even be significant differences in macromolecular estimates across labs using the same protocols [55]. Although differences in extraction efficiency, methods, and standards across studies will influence estimates of the macromolecular pools we do not expect there is any bias in the methods used for specific phyla, so we do not expect this influenced our taxonomic comparison. Culture conditions and technique will also affect macromolecular content and stoichiometry through their influence on physiological state. We reduced this source of variation by focusing our analyses on exponentially growing cultures under nutrient-sufficient conditions but because of the large number of methods and labs involved in this meta-analysis we did not quantify variability due to differences in temperature, carbon dioxide concentration, irradiance and light-dark cycle, salinity, or media used across the studies.

Here we find that fundamental differences in cellular architecture and other biochemical and physiological traits across phyla of microalgae are reflected in bulk differences in macromolecular composition. The phylogenetic differences in macromolecular composition, combined with the elemental stoichiometry of the macromolecules, predict the observed phylum-level differences in C:N from laboratory cultures (Fig 2, Table 4). Macromolecular composition predicts a low molar C:N of 6.0 in the protein-rich Cyanobacteria and the highest C:N of 8.6 in the Dinophyta due to their carbohydrate-rich walls. These estimates of macromolecular and elemental composition should be useful for improving models of phytoplankton biomass and functional group dynamics, and understanding biogeography in C:N:P across environments, especially once variations in macromolecular composition under resource limitation are better understood and quantified. This analysis also provides insight for those looking to identify species with high lipid, protein or carbohydrate composition under nutrient-sufficient exponential growth conditions. The Cyanobacteria and Cryptophyta are most protein-rich, the Dinophyta and Cyanobacteria are the most carbohydrate-rich, and the Haptophyta, Bacillariophyta, and Ochrophyta, especially the Eustigmatophyceae, are most enriched in lipid. Although there are phylum-specific differences in macromolecular composition, much of the variability is at the species level, indicating recent selection pressure on species within phyla has altered macromolecular composition of many species and confirming that bio-prospecting at the species level is likely to yield dividends.

## Supporting Information

S1 File Macromolecular database. Raw data collected from literature sources. See S1 Table for description of table columns.
(CSV)

S1 Table Description of macromolecular database. Description of data in the macromolecular database distributed as “mm-public-database-phylum-level-v1.csv”. The database contains a total of 1562 observations (rows).
(DOCX)
