# Supplementary material for: Phylogenetic Diversity in the Macromolecular Composition of Microalgae
Source: PLoS One. 2016 May 26;11(5):e0155977. doi: 10.1371/journal.pone.0155977 (PMC4882041; doi:10.1371/journal.pone.0155977)
Supplement: S1 Table — Description of data in the macromolecular database distributed as “mm-public-database-phylum-level-v1.csv”. The database contains a total of 1562 observations (rows). (DOCX) [file pone.0155977.s002.docx]

**Supporting Information**

**S1 Table. Description of macromolecular database.** Description of data in the macromolecular database distributed as “mm-public-database-phylum-level-v1.csv”. The database contains a total of 1562 observations (rows).

| **Column name** | **Description** |
| --- | --- |
| Study | An internal sequence number for each study. |
| APhylum, Agenus, Aspecies | Taxonomic information corrected with synonyms from Algaebase. |
| Genus, Species | Taxonomic information from the source publication. |
| Marine | TRUE if the species is found in marine, coastal, or brackish environments or FALSE otherwise. From Algaebase.org and source publication. |
| Freshwater | TRUE if the species is found in freshwater environments or FALSE otherwise. From Algaebase.org and source publication. |
| Exponential.phase | 1 if the samples were taken during the exponential phase of growth, 0 if samples were taken during the early or late stationary phases of growth. |
| Protein.method | An indication of the protein assay method: Protein (including methods quantifying amino acids and peptide residues), N content (as reported in source publication, not as corrected in our analysis), or an unknown method. |
| pg.Protein | Protein content per cell in pg. |
| pg.Lipid | Lipid content per cell in pg. |
| pg.Carbo | Carbohydrate content per cell in pg. |
| pg.Chla | Chlorophyll a content per cell in pg. |
| pg.DNA | DNA content per cell in pg. |
| pg.RNA | RNA content per cell in pg. |
| pg.Ash | Ash content per cell in pg. |
| X..Protein through X..Ash | Same macromolecular content as above, but reported as a percentage of cell dry weight. Any one study may report content in pg, percent or both. If pg content and dry weight (in pg) was available, we computed macromolecular content on a percent dry weight basis. |
| Citation | The full citation for the study. |
| Year | The publication year of the study. |
